# Supplementary material for: Magnetically tunable bidirectional locomotion of a self-assembled nanorod-sphere propeller
Source: Nat Commun. 2018 Apr 25;9:1663. doi: 10.1038/s41467-018-04115-w (PMC5916950; doi:10.1038/s41467-018-04115-w)
Supplement: Supplementary file 1 — Supplementary Information [file 41467_2018_4115_MOESM1_ESM.pdf]

SUPPLEMENTARY FIGURES

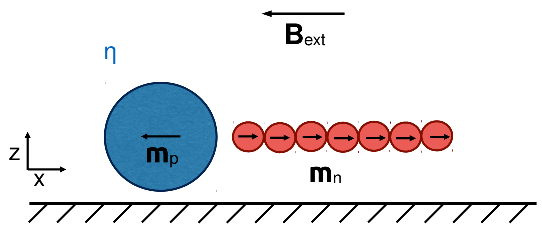

Supplementary Figure 1

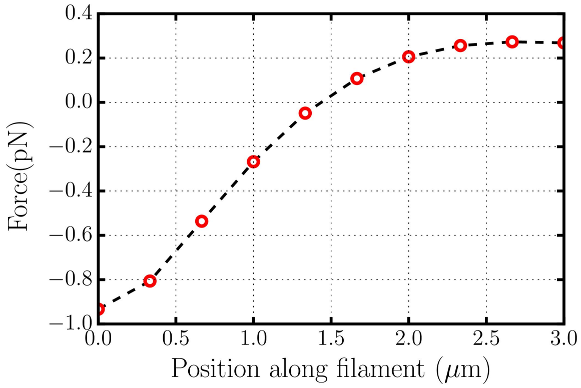

Supplementary Figure 2

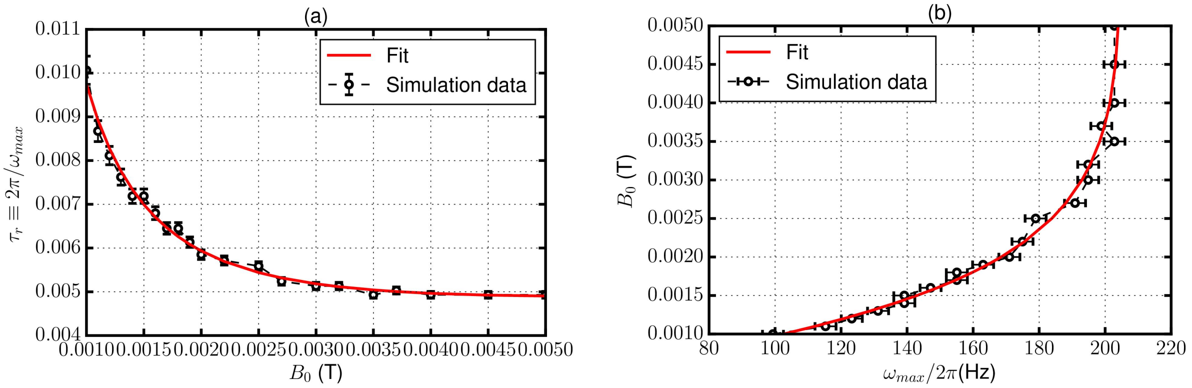

Supplementary Figure 3

SUPPLEMENTARY NOTES

Supplementary Notes 1

we here calculate the hydrodynamic drag on a nanorod after field reversal for $B_0 > B_{\rm ferro}$. For a square-wave modulated field with amplitude $B_0 > B_{\rm ferro}$, as the magnetic field changes polarity, the paramagnetic sphere becomes polarized in the direction of the magnetic field, which is antiparallel to the magnetic moment of the ferromagnet (see Supplementary Figure 1). As a result, the magnetic particles repel each other due to dipole-dipole interaction and separate. Such repulsion generates a hydrodynamic flow which acts on the ferromagnetic nanorod. In our treatment, the paramagnetic sphere is approximated by a point particle and the nanorod as a collection of N-point particles aligned, as far as their hydrodynamic interactions are concerned (see Supplementary Figure 1).

Accordingly, the vertical hydrodynamic drag on particle i of the nanorod is given by

$$F^z_{\rm drag}(\mathbf{r}_i) = 3\pi\eta d[G^{zx}_B(\mathbf{r}_i,\mathbf{r}_p)F^x_p + G^{zz}_B(\mathbf{r}_i,\mathbf{r}_p)F^z_p + \sum_{j\neq i}^{N}(G^{zx}_B(\mathbf{r}_i,\mathbf{r}_j)f^x(\mathbf{r}_j) + G^{zz}_B(\mathbf{r}_i,\mathbf{r}_j)f^z(\mathbf{r}_j))] \quad (1)$$

Here, $G^{\alpha\beta}_B(\mathbf{r}_i,\mathbf{r}_j)$ is the Blake tensor [1], $\mathbf{r}_p$ the position of the paramagnet, F^α_p the force on the paramagnet and $f^\alpha(\mathbf{r}_j)$ the force on the particle j of the ferromagnet. For repulsive dipolar interactions generated by the configuration in Supplementary Figure 1, the vertical drag is nonuniform along the ferromagnetic rod, generating a net torque (see Supplementary Figure 2).

Supplementary Notes 2

We here calculate the dependence of $\omega_{\rm max}$ on the external field. As a consequence of its rotation, the ferromagnetic nanorod experiences a net upward motion due to the interaction with the bounding surface. The relaxation time τ_r that takes the propeller to sediment and reach the minimum energy configuration controls the onset of the propulsion mechanism at high frequencies (at $\omega > \omega_{\rm max} = 2\pi/\tau_r$).

To calculate τ_r we assume that we can split such relaxation into two processes, a sedimentation of the propeller until it reaches the bounding plate and a subsequent alignment of the pair with the external field,

$$\tau_r = \tau_{\rm sed} + \tau_{\rm align}\,. \quad (2)$$

The characteristic sedimentation time is field-independent, given by

$$\tau_{\rm sed} \approx \frac{h6\pi\eta a}{M_p g}\,, \quad (3)$$

where η is the viscosity of the fluid, g the acceleration of gravity, and M_p and a are the mass and radius of the spherical colloid, respectively. h is the distance the sphere is lifted as a result of the rotation of the nanorod which we leave as a fitting constant. Here, we use the fact that the drag and mass of the paramagnetic colloid are much larger than the corresponding values of the nanorod. In sedimenting, the propeller becomes tilted with respect to the horizontal (X-axis), with the free tip of the nanorod directed upwards due to the lower friction per mass of the spherical colloid. We assume that the propeller reaches a stationary value of the orientation θ with the X-axis. The angle θ can be obtained by balancing the torque exerted by the external field on the propeller $T_B \approx m_n B_{\rm ext}\sin\theta$ and the vertical drag force exerted by the fluid as the propeller sediments $T_{\rm hyd} = T^0_{\rm hyd}\cos\theta$. Here, $T^0_{\rm hyd}$ is a constant which depends on the geometry and mass distribution of the propeller. In the cases considered here $T^0_{\rm hyd} \ll m_n B_{\rm ext}$ and

$$\tan\theta \approx 2\tan(\theta/2) \approx T^0_{\rm hyd}/(m_n B_{\rm ext})\,. \quad (4)$$

Once the spherical colloid reaches the bounding plate, the pair reorients to align its magnetic moment with the external field $\boldsymbol{B}_{\rm ext}$. The balance between the torque exerted by the external field and the drag of the fluid on the propeller as it rotates gives

$$\begin{aligned}\tau_{\rm align} &\approx \frac{\xi^p_r}{m_n B_{\rm ext}}\log\left[\tan(\theta/2)\right] + C_1 \\ &\approx \frac{\xi^p_r}{m_n B_{\rm ext}}\log\left[\frac{C}{m_n B_{\rm ext}}\right]\,, \end{aligned} \quad (5)$$

where ξ_r^p is the rotational friction coefficient of the propeller and C_1, C undefined constants.

We have used Supplementary Equations. (2), (3), (5) to fit the results for $\tau_r \equiv 2\pi/\omega_{\max}$ obtained from simulations of the propeller for different values the square-wave field amplitude, see Supplementary Figure 3.

Supplementary References

[1] J. R. Blake, Proc. Gamb. Phil. Soc. **70**, 303 (1971).
[2] Humphrey, W., Dalke, A. and Schulten, K., J. Molec. Graphics, **14**, 33-38 (1996).
