# Supplementary material for: Magnetically tunable bidirectional locomotion of a self-assembled nanorod-sphere propeller
Source: Nat Commun. 2018 Apr 25;9:1663. doi: 10.1038/s41467-018-04115-w (PMC5916950; doi:10.1038/s41467-018-04115-w)
Supplement: Supplementary file 3 — Description of Additional Supplementary Files [file 41467_2018_4115_MOESM3_ESM.pdf]

Description of Additional Supplementary Files

File Name: Supplementary Movie 1
Description: This videoclip illustrates the transportation of the hybrid nanorod-sphere propeller when subjected to an external square wave magnetic modulation in two situations. In the top vidoclip, the prototype moves with the nanorod located on the front of it and dragging the spherical particle. Applied field parameters are field amplitude B0=4.2 mT, driving frequency f = 100 Hz. In the bottom videoclip, the nanorod behaves as a pusher, being located on the back of the colloid. The applied field parameters here are B0 = 8.9 mT, and f = 100 Hz. The two videos correspond to the Fig.1(a) and Fig.1(c) of the article.

File Name: Supplementary Movie 2
Description: The videoclip is illustrating the dynamics of the hybrid nanorod-sphere propeller recorded with a fast CCD camera working at 350 frame per second showing the fast dynamics of the nanorod when reorienting in order to follow the swept of the applied field. The field parameters here are B0=2.6 mT, and f = 4 Hz. The video corresponds to the Fig.2(b) of the article.

File Name: Supplementary Movie 3
Description: Video illustrating the binding/unbinding capability of the hybrid prototype. The latter is initially propelled toward the top by a square wave modulation with amplitude B0 = 3.4 mT and driving frequency f = 20 Hz. After 5.7 s the applied field is switched off and the pair performs simple Brownian motion, while the nanorod is kept assembled to the spherical particle due to its ferromagnetic nature and the attraction with the small induced moment within the particle. 8.9 s later a strong perpendicular magnetic field is used to separate the nanorod from the spherical particle. At the end of the video, the same magnetic field is rotated in the plane in order to approach again the two elements and reform the pair. The video corresponds to the Fig.4(b) of the article.

File Name: Supplementary Movie 4
Description: A colloidal chain composed by 5 particles assembled via attractive dipolar forces and driven by a single nanorod when subjected to a square wave modulation of amplitude B0 = 4.7 mT and frequency f = 20 Hz. The video corresponds to the Fig.4(b) of the article.

File Name: Supplementary Movie 5
Description: Colloidal transport triggered by the rotation of a smaller nanorod of 100 nm diameter. In the top videoclip, the prototype moves with the nanorod acting as a puller. Applied field parameters are B0 = 4.7 mT, f = 20 Hz. In the bottom videoclip, the nanorod behave as a pusher, being located on the back of the colloid. The applied field parameters here are B0 =5.8 mT, and f = 20 Hz. The two videos correspond to the Fig.4(d)

File Name: Supplementary Movie 6
Description: Video obtained for an amplitude B0 = 3mT (B0 < Bc2) and f = 100 Hz. It shows the rotation of the ferromagnetic nanorod in a clockwise direction, which induces a translation due to the hydrodynamic interaction with the plane. In this case, the

File Name: Supplementary Movie 7
Description: Video obtained for an amplitude B0 = 7mT (B0 > Bc2) and f = 100 Hz. It shows the rotation of the ferromagnetic nanorod in a counterclockwise direction, resulting in the translocation of the spherical paramagnetic colloid located on its left.
